# Physical Activity and Health: Social Psychology Perspective

**DOI:** 10.3390/bs13040286

**Published:** 2023-03-27

**Authors:** Rogério César Fermino

**Affiliations:** Research Group on Environment, Physical Activity, and Health, Postgraduate Program in Physical Education, Federal University of Technology-Paraná, Curitiba 81310-900, Brazil; rogeriofermino@utfpr.edu.br

Physical inactivity is a significant global health problem. If there is no reduction in physical inactivity worldwide, it is estimated that around 500 million new cases of chronic diseases could occur by 2030 [1]. Of these, 74% of cases will occur in low-and middle-income countries. In addition to the burden on countries’ public health systems, the economic impact could reach USD 300 billion [1]. The world’s population is exposed to a high risk of chronic diseases due to physical inactivity; 28% of adults do not reach the guidelines for physical activity [2].

Physical activity is a behavior of great significance in the context of societies globally. Promoting an active lifestyle has been used to improve health standards and quality of life, in which physical activity has been widely encouraged because of its physical and psychosocial benefits [3]. The research contributes to the broad understanding of behavior for developing new interventions that promote health.

An important question is: what must we do to make people practice more physical activity? Physically active behavior is complex and determined by questions at some levels: individual (biological and psychological), interpersonal (social support and cultural norms and practices), environment (social, built, and natural environment), regional or national policy (e.g., urban planning and national physical activity plans), and global (e.g., economic development, and social and cultural norms) [4,5]. A multidetermined behavior requires complex approaches and efforts to comprehend.

This Special Issue aims at advancing the literature on “Physical Activity and Health: Social Psychology Perspective” from interdisciplinary perspectives. Therefore, we welcome theoretical or empirical contributions that broaden the knowledge of those factors as social mechanisms of physical activity promotion. 
